# Supplementary material for: Discrimination of Adzuki Bean (Vigna angularis) Geographical Origin by Targeted and Non-Targeted Metabolite Profiling with Gas Chromatography Time-of-Flight Mass Spectrometry
Source: Metabolites. 2020 Mar 17;10(3):112. doi: 10.3390/metabo10030112 (PMC7143473; doi:10.3390/metabo10030112)
Supplement: Supplementary file 1 [file metabolites-10-00112-s001.zip › Metabolites_supplementary materials_FV.pdf]

Supplementary figures

Discrimination of geographical origin of adzuki bean (*Vigna angularis*) based on targeted and non-targeted metabolite profiling by using gas chromatography-time-of-flight mass spectrometry

Tae Jin Kim [1,†], Jeong Gon Park [1,†], Soon Kil Ahn [1], Kil Won Kim [1], Jaehyuk Choi [1], Hyun Young Kim [2], Woo Duck Seo [2,*], and Jae Kwang Kim [1,*]

[1] Division of Life Sciences, College of Life Sciences and Bioengineering, Incheon National Univ., Incheon, 22012, Republic of Korea; f91gd@inu.ac.kr (T.J.K); ToLuck@inu.ac.kr (J.G.P); skahn@inu.ac.kr (S.K.A); kilwon@inu.ac.kr (K.W.K); jaehyukc@inu.ac.kr (J.C); kjkpj@inu.ac.kr (K.J.K.)

[2] Division of Crop Foundation, National Institute of Crop Science, Rural Development Administration, Wanju, Jeonbuk, 55365, Republic of Korea; hykim@korea.kr (H.Y.K); swd2002@korea.kr (W.D.S)

* Correspondence: swd2002@korea.kr (W.D.S); Tel.: +82-63-238-5305; kjkpj@inu.ac.kr (K.J.K.); Tel.: +82-32-835-8241

† These authors contributed equally to this work.

Supplementary figures

Figure S1. *PCA score plots of targeted and non-targeted metabolite profiling with UV-scaling and pareto-scaling normalization. A (Target and UV-scaling), B (Non-target and UV-scaling), C (Target and pareto-scaling), D (Non-target and pareto-scaling).*

Figure S2. *PCA loading plots of targeted and non-targeted metabolite profiling with UV-scaling and pareto-scaling normalization. A (Target and UV-scaling), B (Non-target and UV-scaling), C (Target and pareto-scaling), D (Non-target and pareto-scaling).*

Figure S3. *OPLS-DA loading plots of targeted and non-targeted metabolite profiling with UV-scaling and pareto-scaling normalization. A (Target and UV-scaling), B (Non-target and UV-scaling), C (Target and pareto-scaling), D (Non-target and pareto-scaling).*

Figure S4. *OPLS-DA VIP (variables importance in the projection) plot of targeted and non-targeted metabolite profiling with UV-scaling and pareto-scaling normalization. A (Target and UV-scaling), B (Non-target and UV-scaling), C (Target and pareto-scaling), D (Non-target and pareto-scaling).*

Figure S5. *Permutation test of OPLS-DA of non-targeted metabolite profiling with pareto-scaling normalization The number of permutations for the permutation test is 200.*

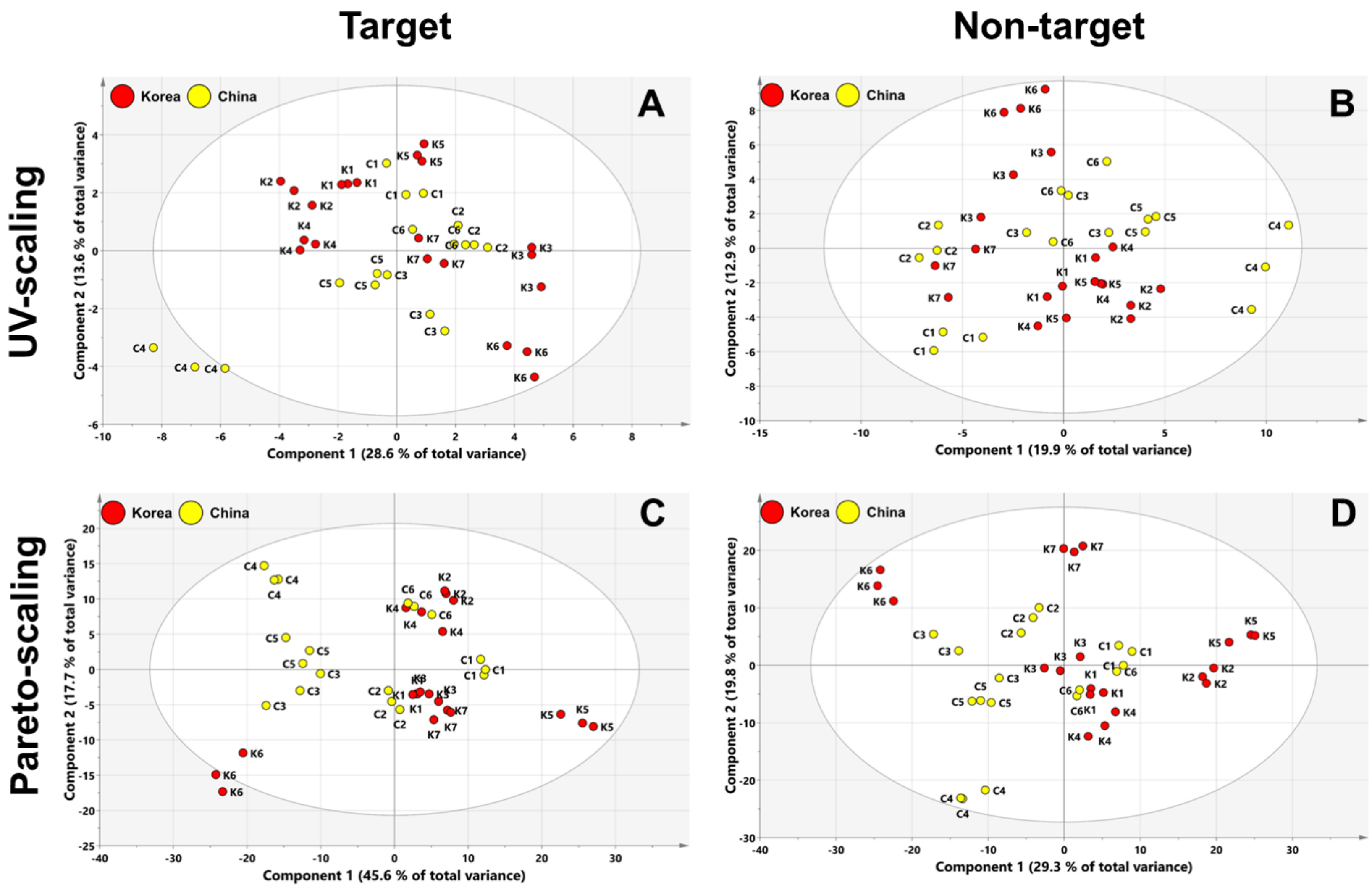

Figure S1. PCA score plots of targeted and non-targeted metabolite profiling with UV-scaling and pareto-scaling normalization. A (Target and UV-scaling), B (Non-target and UV-scaling), C (Target and pareto-scaling), D (Non-target and pareto-scaling). K1, Korea 1; K2, Korea 2; K3, Korea 3; K4, Korea 4; K5, Korea 5; K6, Korea 6; K7, Korea 7; C1, China 1; C2, China 2; C3, China 3; C4, China 4; C5, China 5; C6, China 6.

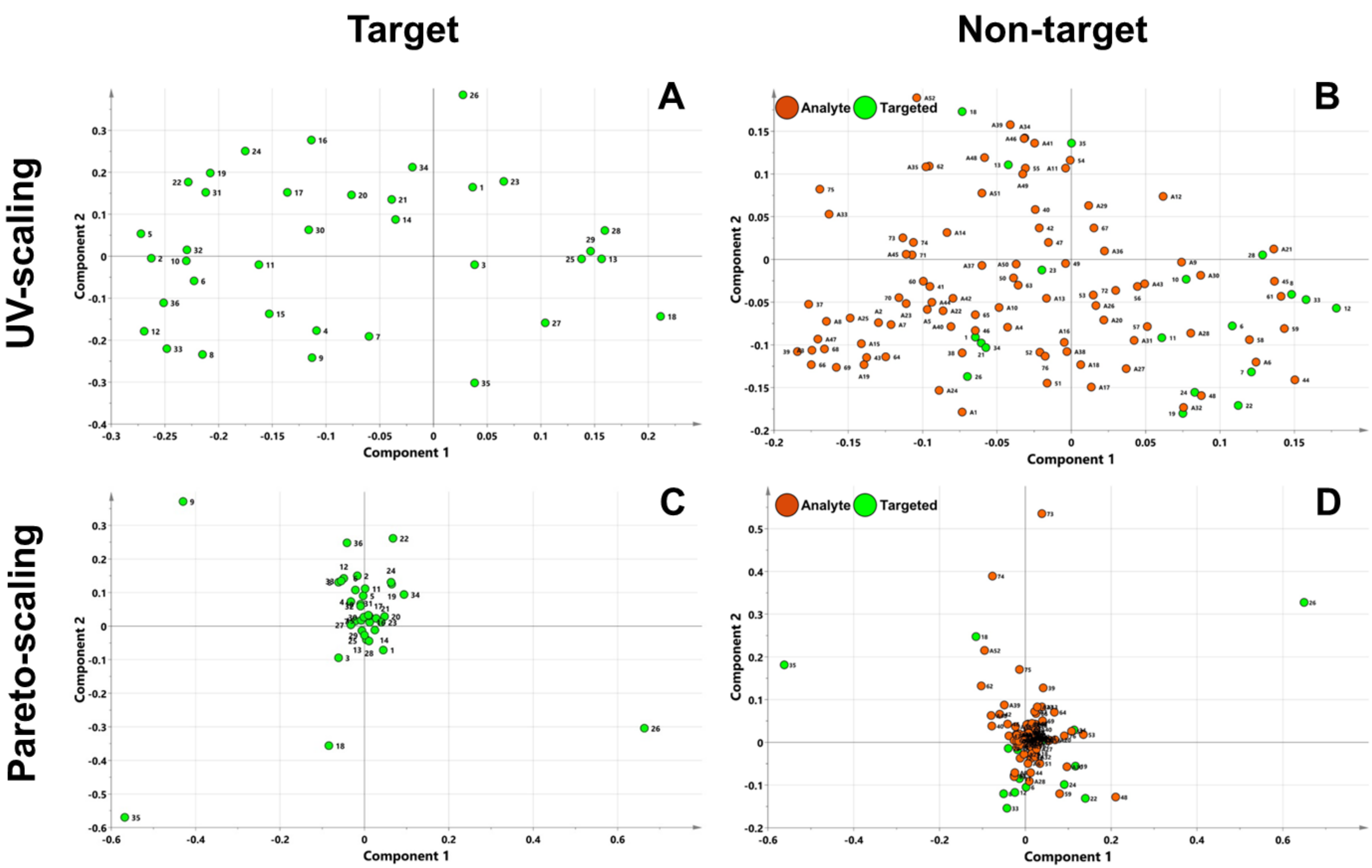

Figure S2. PCA loading plots of targeted and non-targeted metabolite profiling with UV-scaling and pareto-scaling normalization. A (Target and UV-scaling), B (Non-target and UV-scaling), C (Target and pareto-scaling), D (Non-target and pareto-scaling).

(Continued figure S2). 1.Lactic acid; 2. Alanine; 3. Oxalic acid; 4. Glycolic acid; 5. Valine; 6. Urea; 7. Serine; 8. Ethanolamine; 9. Phosphoric acid; 10. Isolucine; 11. Proline; 12. Glycine; 13. Succinic acid; 14. Glyceric acid; 15. Fumaric acid; 16. Threonine; 17. β-Alanine; 18. Malic acid; 19. Aspartic acid; 20. Pyroglutamic acid; 21. Threonic acid; 22. Glutamic acid; 23. Phenylalanine; 24. Asparagine; 25. Shikimic acid; 26. Citric acid; 27. Fructose; 28. Galactose; 29. Glucose; 30. Mannitol; 31. Lysine; 32. Tyrosine; 33. Inositol; 34. Tryptophan; 35. Sucrose; 36. Raffinose; 37. Butanedioic acid; 38. Ethylene glycol; 39. 1,3-Propanediol; 40. Propane; 41. Hydroxylamine; 42. N,O,O-tri(trimethylsilyl)-1-c(13)-N-carboxy-glycine; 43. 1,4-Butanediol; 44. Norvaline; 45. Glycerol; 46. 5-Methoxy-3-chromanol; 47. Propanoic acid; 48. Pipecolic acid; 49. L-Aspartic acid; 50. D-(-)-Erythrose; 51. 2-Methoxy-3-methoxycarbonyl-1,3-pentadiene; 52. Suberylglycine; 53. L-(+)-Tartaric acid; 54. D-Ribose; 55. D-Galactose; 56. Allantoin; 57. L-Histidine; 58. 3-(2-fluorophenyl)spiro(oxirane-2,2'-norbornane); 59. D-Pinitol; 60. D-(+)-Talopyranose; 61. Ribonic acid, 2,3,4,5-tetrakis-O-(trimethylsilyl)-, trimethylsilyl ester; 62. Galacturonic acid; 63. Pentadecanoic acid; 64. Tris(trimethylsilyl)amine; 65. Ethyl D-glucopyranoside; 66. Cyclohexanecarboxylic acid; 67. Bis(2-ethylhexyl) phthalate; 68. (2,3-Diphenylcyclopropyl)methyl phenyl sulfoxide1; 69. (2,3-Diphenylcyclopropyl)methyl phenyl sulfoxide2; 70. Tetracyclo[4.3.0.0(2,7)]nonane-7,7,8,8-tetracarbonitrile; 71. Methyl galactoside (1S,2R,3S,4R,5R)-; 72. D-Galactoside; 73. D-Glucopyranose; 74. D-Galactose 1; 75. D-Galactose2; 76. Dimethyl hexopyranosiduronate; A1 ~ 52. Analyte.

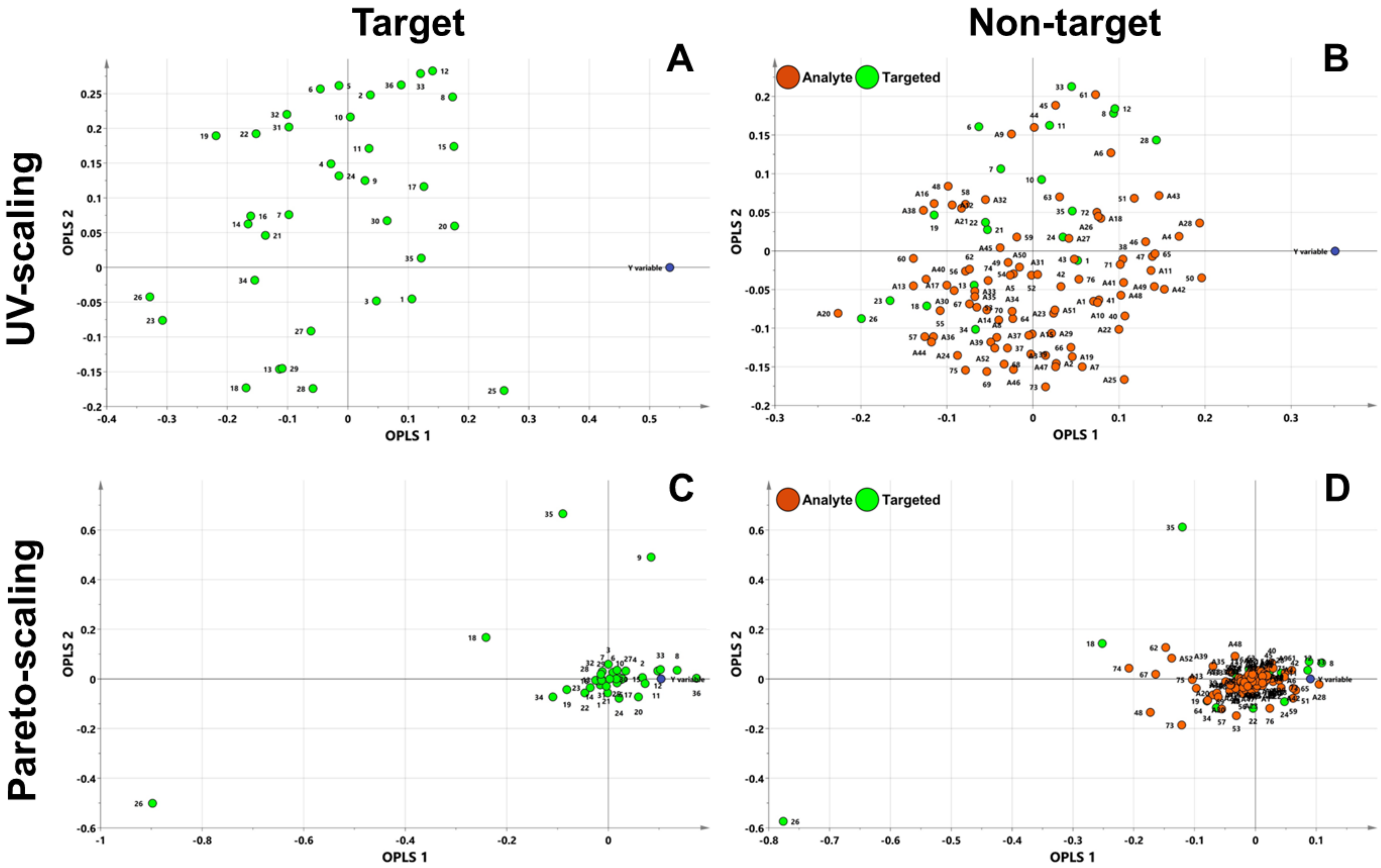

Figure S3. OPLS-DA loading plots of targeted and non-targeted metabolite profiling with UV-scaling and pareto-scaling normalization. A (Target and UV-scaling), B (Non-target and UV-scaling), C (Target and pareto-scaling), D (Non-target and pareto-scaling).

(Continued figure S3). 1.Lactic acid; 2. Alanine; 3. Oxalic acid; 4. Glycolic acid; 5. Valine; 6. Urea; 7. Serine; 8. Ethanolamine; 9. Phosphoric acid; 10. Isolucine; 11. Proline; 12. Glycine; 13. Succinic acid; 14. Glyceric acid; 15. Fumaric acid; 16. Threonine; 17. β-Alanine; 18. Malic acid; 19. Aspartic acid; 20. Pyroglutamic acid; 21. Threonic acid; 22. Glutamic acid; 23. Phenylalanine; 24. Asparagine; 25. Shikimic acid; 26. Citric acid; 27. Fructose; 28. Galactose; 29. Glucose; 30. Mannitol; 31. Lysine; 32. Tyrosine; 33. Inositol; 34. Tryptophan; 35. Sucrose; 36. Raffinose; 37. Butanedioic acid; 38. Ethylene glycol; 39. 1,3-Propanediol; 40. Propane; 41. Hydroxylamine; 42. N,O,O-tri(trimethylsilyl)-1-c(13)-N-carboxy-glycine; 43. 1,4-Butanediol; 44. Norvaline; 45. Glycerol; 46. 5-Methoxy-3-chromanol; 47. Propanoic acid; 48. Pipecolic acid; 49. L-Aspartic acid; 50. D-(-)-Erythrose; 51. 2-Methoxy-3-methoxycarbonyl-1,3-pentadiene; 52. Suberylglycine; 53. L-(+)-Tartaric acid; 54. D-Ribose; 55. D-Galactose; 56. Allantoin; 57. L-Histidine; 58. 3-(2-fluorophenyl)spiro(oxirane-2,2'-norbornane); 59. D-Pinitol; 60. D-(+)-Talopyranose; 61. Ribonic acid, 2,3,4,5-tetrakis-O-(trimethylsilyl)-, trimethylsilyl ester; 62. Galacturonic acid; 63. Pentadecanoic acid; 64. Tris(trimethylsilyl)amine; 65. Ethyl D-glucopyranoside; 66. Cyclohexanecarboxylic acid; 67. Bis(2-ethylhexyl) phthalate; 68. (2,3-Diphenylcyclopropyl)methyl phenyl sulfoxide1; 69. (2,3-Diphenylcyclopropyl)methyl phenyl sulfoxide2; 70. Tetracyclo[4.3.0.0(2,7)]nonane-7,7,8,8-tetracarbonitrile; 71. Methyl galactoside (1S,2R,3S,4R,5R)-; 72. D-Galactoside; 73. D-Glucopyranose; 74. D-Galactose 1; 75. D-Galactose2; 76. Dimethyl hexopyranosiduronate; A1 ~ 52. Analyte.

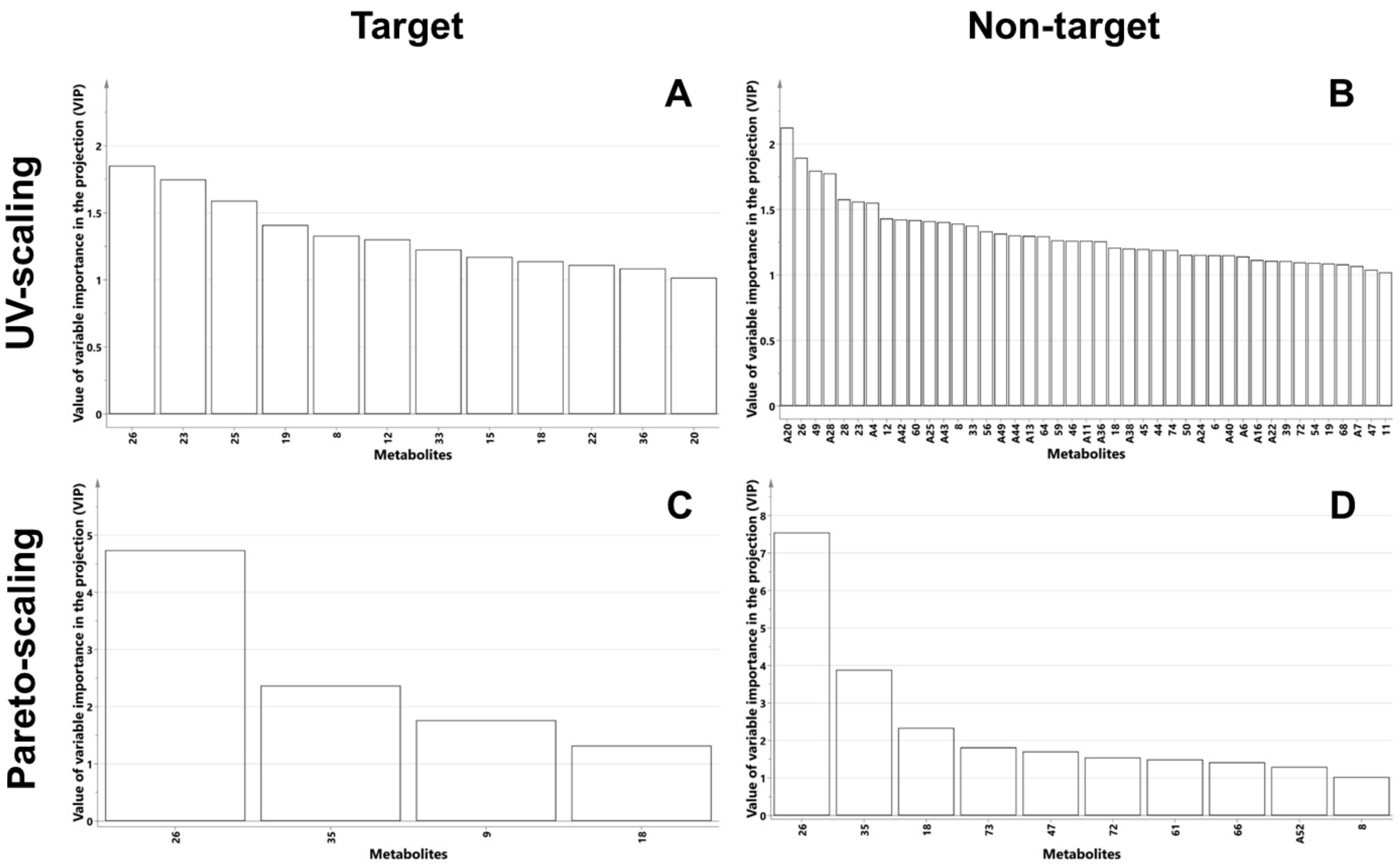

Figure S4. OPLS-DA VIP (variables importance in the projection) plot of targeted and non-targeted metabolite profiling with UV-scaling and pareto-scaling normalization. A (Target and UV-scaling), B (Non-target and UV-scaling), C (Target and pareto-scaling), D (Non-target and pareto-scaling).

(Continued figure S4). 1.Lactic acid; 2. Alanine; 3. Oxalic acid; 4. Glycolic acid; 5. Valine; 6. Urea; 7. Serine; 8. Ethanolamine; 9. Phosphoric acid; 10. Isolucine; 11. Proline; 12. Glycine; 13. Succinic acid; 14. Glyceric acid; 15. Fumaric acid; 16. Threonine; 17. β-Alanine; 18. Malic acid; 19. Aspartic acid; 20. Pyroglutamic acid; 21. Threonic acid; 22. Glutamic acid; 23. Phenylalanine; 24. Asparagine; 25. Shikimic acid; 26. Citric acid; 27. Fructose; 28. Galactose; 29. Glucose; 30. Mannitol; 31. Lysine; 32. Tyrosine; 33. Inositol; 34. Tryptophan; 35. Sucrose; 36. Raffinose; 37. Butanedioic acid; 38. Ethylene glycol; 39. 1,3-Propanediol; 40. Propane; 41. Hydroxylamine; 42. N,O,O-tri(trimethylsilyl)-1-c(13)-N-carboxy-glycine; 43. 1,4-Butanediol; 44. Norvaline; 45. Glycerol; 46. 5-Methoxy-3-chromanol; 47. Propanoic acid; 48. Pipecolic acid; 49. L-Aspartic acid; 50. D-(-)-Erythrose; 51. 2-Methoxy-3-methoxycarbonyl-1,3-pentadiene; 52. Suberylglycine; 53. L-(+)-Tartaric acid; 54. D-Ribose; 55. D-Galactose; 56. Allantoin; 57. L-Histidine; 58. 3-(2-fluorophenyl)spiro(oxirane-2,2'-norbornane); 59. D-Pinitol; 60. D-(+)-Talopyranose; 61. Ribonic acid, 2,3,4,5-tetrakis-O-(trimethylsilyl)-, trimethylsilyl ester; 62. Galacturonic acid; 63. Pentadecanoic acid; 64. Tris(trimethylsilyl)amine; 65. Ethyl D-glucopyranoside; 66. Cyclohexanecarboxylic acid; 67. Bis(2-ethylhexyl) phthalate; 68. (2,3-Diphenylcyclopropyl)methyl phenyl sulfoxide1; 69. (2,3-Diphenylcyclopropyl)methyl phenyl sulfoxide2; 70. Tetracyclo[4.3.0.0(2,7)]nonane-7,7,8,8-tetracarbonitrile; 71. Methyl galactoside (1S,2R,3S,4R,5R)-; 72. D-Galactoside; 73. D-Glucopyranose; 74. D-Galactose 1; 75. D-Galactose2; 76. Dimethyl hexopyranosiduronate; A1 ~ 52. Analyte.

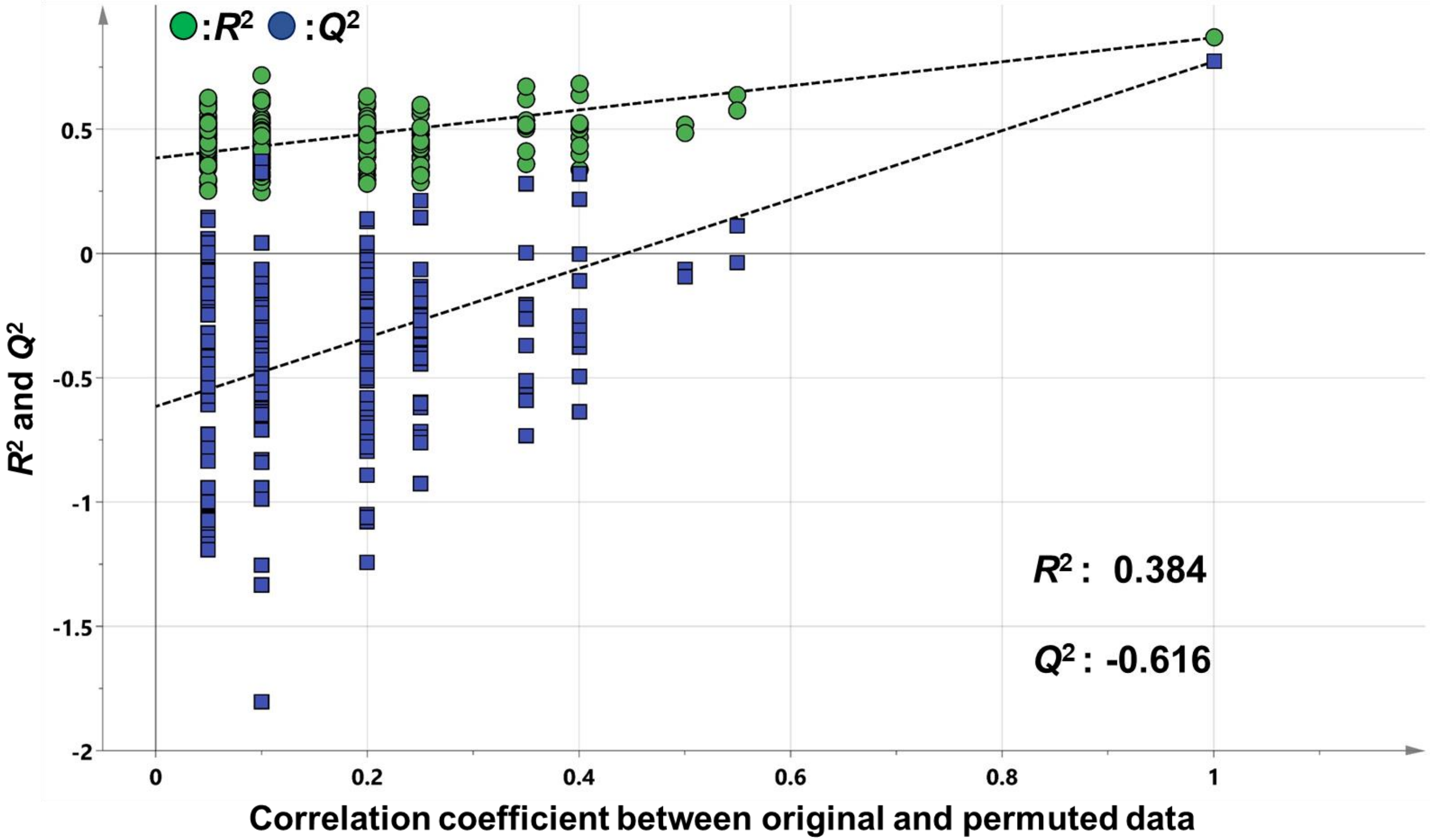

Figure S5. Permutation test of OPLS-DA of non-targeted metabolite profiling with pareto-scaling normalization The number of permutations for the permutation test is 200.

Supplementary tables

Discrimination of geographical origin of adzuki bean (*Vigna angularis*) based on targeted and non-targeted metabolite profiling by using gas chromatography-time-of-flight mass spectrometry

Tae Jin Kim [1,†], Jeong Gon Park [1,†], Soon Kil Ahn [1], Kil Won Kim [1], Jaehyuk Choi [1], Hyun Young Kim [2], Woo Duck Seo [2,*], and Jae Kwang Kim [1,*]

[1] Division of Life Sciences, College of Life Sciences and Bioengineering, Incheon National Univ., Incheon, 22012, Republic of Korea; f91gd@inu.ac.kr (T.J.K); ToLuck@inu.ac.kr (J.G.P); skahn@inu.ac.kr (S.K.A); kilwon@inu.ac.kr (K.W.K); jaehyukc@inu.ac.kr (J.C); kjkpj@inu.ac.kr (K.J.K.)

[2] Division of Crop Foundation, National Institute of Crop Science, Rural Development Administration, Wanju, Jeonbuk, 55365, Republic of Korea; hykim@korea.kr (H.Y.K); swd2002@korea.kr (W.D.S)

* Correspondence: swd2002@korea.kr (W.D.S); Tel.: +82-63-238-5305; kjkpj@inu.ac.kr (K.J.K.); Tel.: +82-32-835-8241

† These authors contributed equally to this work.

Supplementary tables

Table S1. *Composition and content (ratio/g) of hydrophilic compounds by using targeted metabolite profiling of adzuki bean (Vigna angularis) cultivated in Korea.*

Table S2. *Composition and content (ratio/g) of hydrophilic compounds by using targeted metabolite profiling of adzuki bean (Vigna angularis) cultivated in China.*

Table S3. *Composition and content (ratio/g) of hydrophilic compounds by using non-targeted metabolite profiling, which matched with targeted metabolite profiling, of adzuki bean (Vigna angularis) cultivated in Korea.*

Table S4. *Composition and content (ratio/g) of hydrophilic compounds by using non-targeted metabolite profiling, which matched with targeted metabolite profiling, of adzuki bean (Vigna angularis) cultivated in China*

Table S5. *PLS-DA loading and VIP of variables from targeted metabolite profiling with UV-scaling.*

Table S6. *PLS-DA loading and VIP of variables from non-targeted metabolite profiling with UV-scaling.*

Table S7. *PLS-DA loading and VIP of variables from targeted metabolite profiling with pareto-scaling.*

Table S8. *PLS-DA loading and VIP of variables from non-targeted metabolite profiling with pareto-scaling*

Table S9. *Relative retention times (RRT) and mass spectral data of hydrophilic compounds as trimethylsilyl derivatives.*

Table S1. Composition and content (ratio/g) of hydrophilic compounds by using targeted metabolite profiling of adzuki bean (*Vigna angularis*) cultivated in Korea.

CI[a]	Compound	K1	K2	K3	K4	K5	K6	K7
1	Lactic acid	15.79±8.85	3.66±0.77	10.70±1.51	8.51±0.37	10.02±1.29	5.50±1.30	7.88±1.67
2	Alanine	5.77±0.60	8.45±0.90	3.02±1.10	6.71±1.56	6.04±0.89	2.02±0.64	2.84±0.70
3	Oxalic acid	50.68±13.63	44.75±1.97	39.28±6.18	38.72±5.29	39.75±1.87	45.65±15.53	38.73±2.40
4	Glycolic acid	5.57±1.15	7.18±0.16	8.74±1.89	12.95±0.88	5.41±0.47	7.97±0.40	5.69±0.68
5	Valine	4.38±0.52	4.91±0.45	2.21±0.79	4.62±0.58	3.79±0.32	1.82±0.39	3.06±0.45
6	Urea	8.91±1.66	7.11±0.18	3.06±0.16	13.90±0.64	4.54±0.32	5.77±0.74	3.78±0.33
7	Serine	0.55±0.11	1.69±0.21	0.40±0.35	0.79±0.07	0.42±0.06	1.28±0.30	1.11±0.37
8	Ethanolamine	1.92±0.16	2.02±0.18	1.72±0.41	4.65±0.79	1.76±0.13	2.77±0.54	2.98±0.46
9	Phosphoric acid	178.27±1.19	273.82±14.58	144.33±2.85	223.18±4.56	71.85±2.40	252.36±14.63	108.98±1.23
10	Isoleucine	1.33±0.13	2.21±0.17	0.81±0.25	2.35±0.39	1.28±0.11	0.66±0.25	1.41±0.25
11	Proline	6.30±0.54	7.39±0.52	9.85±3.10	13.70±3.41	6.74±0.17	3.44±0.90	7.94±1.18
12	Glycine	6.13±0.41	7.70±0.63	3.48±0.30	8.12±1.58	5.33±0.55	5.33±0.68	5.11±0.16
13	Succinic acid	11.75±0.35	9.63±0.44	18.70±0.83	9.44±0.88	9.28±0.45	10.84±0.54	9.15±0.25
14	Glyceric acid	1.42±0.11	1.43±0.09	1.95±0.22	1.61±0.18	2.81±0.13	1.20±0.06	2.27±0.27
15	Fumaric acid	0.68±0.06	0.45±0.04	0.70±0.06	0.56±0.10	0.49±0.09	0.34±0.07	0.70±0.17
16	Threonine	1.16±0.01	1.23±0.19	0.64±0.18	0.77±0.09	1.01±0.13	0.62±0.21	0.73±0.04
17	β-Alanine	2.74±0.62	1.66±0.35	1.39±0.08	3.26±0.24	2.00±0.11	1.47±0.17	1.33±0.18
18	Malic acid	40.10±1.19	54.03±1.25	79.46±0.91	36.25±3.89	49.75±2.21	88.26±1.47	64.36±1.44
19	Aspartic acid	24.90±0.92	32.99±0.79	16.1±1.93	22.40±1.16	21.66±0.46	11.69±1.86	25.18±1.48
20	Pyroglutamic acid	10.49±0.13	5.65±0.20	8.85±0.80	13.70±0.18	13.03±0.41	3.92±0.36	8.18±0.86
21	Threonic acid	5.39±0.26	5.52±0.07	6.30±0.18	7.35±0.19	5.48±0.35	4.52±0.28	6.12±0.67
22	Glutamic acid	44.38±0.61	67.57±2.39	28.67±1.20	36.74±1.83	38.78±1.38	21.74±0.96	34.69±0.85
23	Phenylalanine	2.19±0.12	3.78±0.22	4.55±0.48	3.45±0.06	3.99±0.11	2.37±0.21	2.78±0.21
24	Asparagine	16.27±0.17	11.53±0.24	1.60±0.35	7.91±0.88	11.64±0.62	1.39±0.15	3.22±0.62
25	Shikimic acid	0.75±0.08	0.65±0.05	0.80±0.07	0.55±0.06	0.57±0.05	0.90±0.08	0.62±0.10
26	Citric acid	577.93±3.91	601.50±12.75	501.94±18.97	547.77±37.01	704.56±31.02	436.82±21.10	550.76±5.77
27	Fructose	5.42±1.37	10.31±2.80	17.51±3.85	11.02±2.26	9.11±0.98	11.55±3.27	7.00±1.33
28	Galactose	7.56±0.61	6.16±1.04	8.02±2.34	5.27±1.06	6.76±1.42	6.91±1.39	5.50±0.24
29	Glucose	2.84±0.42	2.40±0.18	3.46±0.67	2.29±0.18	2.61±0.40	2.65±0.50	2.31±0.10

(*Continued next page*)

(*Table S1., continued*)

CI[a]	Compound	K1	K2	K3	K4	K5	K6	K7
30	Mannitol	0.72±0.16	1.13±0.06	0.53±0.13	0.49±0.28	0.44±0.14	0.30±0.06	0.37±0.16
31	Lysine	2.33±0.05	1.97±0.13	0.63±0.08	1.32±0.12	1.78±0.20	1.13±0.22	0.89±0.07
32	Tyrosine	4.02±0.17	5.09±0.09	1.85±0.18	3.15±0.31	2.54±0.08	2.69±0.32	2.77±0.03
33	Inositol	5.82±0.47	6.07±0.67	4.69±0.08	9.20±0.25	3.98±0.35	4.09±0.22	5.20±0.17
34	Tryptophan	15.35±3.64	39.28±2.03	18.72±4.54	24.84±1.14	7.88±0.29	15.26±1.38	17.32±5.35
35	Sucrose	322.58±2.55	200.17±6.68	216.75±5.73	224.99±8.39	193.68±1.32	473.18±37.99	274.57±17.04
36	Raffinose	49.51±1.15	48.80±1.30	38.52±0.79	46.82±0.97	38.90±0.27	33.07±2.14	54.50±1.63

[a] CI, compound index

Table S2. Composition and content (ratio/g) of hydrophilic compounds by using targeted metabolite profiling of adzuki bean (*Vigna angularis*) cultivated in China.

CI[a]	Compound	C1	C2	C3	C4	C5	C6
1	Lactic acid	14.58±5.56	15.17±3.94	7.70±3.74	7.57±3.14	7.31±1.96	5.86±1.92
2	Alanine	4.56±0.86	3.00±0.14	4.61±2.47	10.92±2.05	9.75±3.44	4.51±0.62
3	Oxalic acid	41.83±0.86	48.51±3.88	75.21±69.99	34.15±0.67	39.05±3.61	45.99±10.62
4	Glycolic acid	8.03±0.20	4.40±0.19	8.36±1.90	9.89±0.87	7.06±1.14	6.60±1.18
5	Valine	3.00±0.72	2.55±0.23	2.87±1.09	5.55±0.81	5.49±0.45	2.68±0.17
6	Urea	5.01±1.20	3.22±0.15	5.91±0.88	10.64±0.79	4.16±0.48	4.51±0.14
7	Serine	0.23±0.06	0.53±0.23	1.24±0.48	1.03±0.07	0.64±0.06	0.89±0.04
8	Ethanolamine	3.00±0.43	1.96±0.14	2.15±0.60	17.05±0.95	2.87±0.16	2.33±0.28
9	Phosphoric acid	104.39±0.78	156.52±5.89	225.49±13.48	226.27±5.08	219.00±8.90	185.98±9.45
10	Isoleucine	1.00±0.16	1.12±0.26	1.40±0.30	2.07±0.10	2.81±0.08	1.19±0.11
11	Proline	7.99±1.80	8.21±0.90	6.29±3.00	12.07±1.30	10.89±0.24	4.90±0.37
12	Glycine	5.81±0.23	4.38±0.41	6.69±0.88	14.94±0.62	7.76±0.47	6.49±0.33
13	Succinic acid	9.80±0.76	9.66±0.28	10.26±0.54	8.87±0.74	9.78±0.14	11.58±0.54
14	Glyceric acid	1.45±0.34	1.37±0.17	1.59±0.15	2.11±0.25	1.24±0.26	1.20±0.19
15	Fumaric acid	0.65±0.10	0.68±0.13	0.69±0.04	1.23±0.12	0.54±0.13	0.36±0.10
16	Threonine	0.76±0.14	0.81±0.23	0.67±0.14	0.68±0.10	1.03±0.05	0.73±0.04
17	β-Alanine	2.68±0.49	2.46±0.33	1.67±0.56	2.22±0.39	2.10±0.12	2.13±0.11
18	Malic acid	36.70±0.63	49.35±2.14	56.04±1.69	29.70±1.51	57.51±4.54	35.64±2.88
19	Aspartic acid	19.46±2.93	16.62±0.83	21.11±2.64	22.65±1.75	18.91±0.85	15.77±0.98
20	Pyroglutamic acid	12.82±0.61	14.17±1.21	10.17±1.31	11.39±0.67	10.52±0.52	7.68±0.38
21	Threonic acid	6.22±0.45	5.34±0.35	5.67±0.50	5.19±0.78	5.20±0.29	5.36±0.21
22	Glutamic acid	41.88±2.51	26.64±1.76	36.02±2.02	46.46±2.53	38.98±0.30	34.37±1.85
23	Phenylalanine	2.57±0.14	2.39±0.18	2.57±0.07	2.27±0.29	2.31±0.11	3.55±0.07
24	Asparagine	12.69±1.12	3.42±0.55	6.82±0.52	10.15±0.47	7.70±1.17	13.11±1.35
25	Shikimic acid	0.94±0.20	1.62±0.49	0.90±0.20	0.54±0.12	0.91±0.18	1.14±0.47
26	Citric acid	545.27±13.67	489.29±28.07	433.65±11.00	346.59±23.61	391.94±16.03	455.10±38.07
27	Fructose	5.82±0.80	6.88±2.09	11.51±1.75	9.49±1.59	11.18±1.88	10.76±2.04
28	Galactose	4.87±0.97	7.70±3.29	6.47±0.10	4.29±0.85	5.77±1.01	7.88±1.69
29	Glucose	1.77±0.21	2.65±0.96	2.60±0.28	1.89±0.20	2.73±0.31	2.67±0.58

(*Continued next page*)

(*Table S2., continued*)

CI[a]	Compound	C1	C2	C3	C4	C5	C6
30	Mannitol	0.52±0.09	0.93±0.11	0.76±0.11	0.74±0.08	0.63±0.12	0.53±0.06
31	Lysine	1.20±0.07	0.92±0.14	0.97±0.15	1.93±0.13	1.16±0.03	1.38±0.15
32	Tyrosine	2.27±0.19	2.56±0.14	3.10±0.36	4.02±0.26	2.92±0.10	2.77±0.21
33	Inositol	4.06±0.38	4.31±0.57	6.50±0.67	15.16±0.59	6.25±0.51	4.25±0.43
34	Tryptophan	37.38±4.76	15.39±1.09	7.24±2.77	5.07±0.20	13.56±1.31	17.55±1.18
35	Sucrose	216.00±13.28	281.02±16.94	356.19±22.88	310.84±12.59	317.95±7.43	167.45±9.41
36	Raffinose	42.09±1.10	39.09±1.58	42.99±2.21	72.53±3.99	45.08±1.96	46.28±2.91

[a] CI, compound index

Table S3. Composition and content (ratio/g) of hydrophilic compounds by using non-targeted metabolites profiling, which matched with targeted metabolite profiling, of adzuki bean (*Vigna angularis*) cultivated in Korea.

CI[a]	Compound	K1	K2	K3	K4	K5	K6	K7
1	Lactic acid	14.84±10.06	1.09±1.89	8.17±1.07	6.89±0.26	7.71±1.22	4.60±0.99	6.54±1.09
6	Urea	9.07±1.59	7.20±0.19	3.07±0.17	13.87±0.57	4.65±0.39	5.87±0.85	3.89±0.42
7	Serine	2.06±0.37	1.37±1.20	1.93±0.66	5.24±0.56	0.62±1.07	3.02±0.81	3.18±0.42
8	Ethanolamine	0.46±0.80	2.24±0.14	0.28±0.48	2.50±0.39	1.50±0.27	ND[b]	0.54±0.93
10	Isoleucine	6.24±0.44	7.37±0.56	9.88±3.09	13.72±3.47	6.73±0.13	2.68±2.36	7.95±1.12
11	Proline	6.17±0.34	7.79±0.50	3.57±0.29	8.15±1.43	5.37±0.44	5.44±0.70	5.23±0.08
12	Glycine	11.38±0.38	9.60±0.23	18.94±0.78	9.49±0.77	9.46±0.51	10.92±0.43	9.15±0.28
13	Succinic acid	1.45±0.20	2.91±0.08	1.30±0.42	2.05±0.18	1.95±0.29	0.97±0.39	1.75±0.11
18	Malic acid	39.97±1.45	54.04±1.24	79.52±0.93	36.55±3.91	49.66±2.33	88.80±0.36	64.56±1.41
19	Aspartic acid	34.57±0.72	47.54±1.14	23.08±2.92	32.24±2.17	30.03±1.07	16.73±3.23	35.29±2.69
21	Threonic acid	1.51±0.16	1.46±0.10	1.72±0.19	2.08±0.14	1.48±0.16	1.32±0.09	1.88±0.14
22	Glutamic acid	44.18±0.63	67.47±2.29	28.59±1.27	36.73±1.90	38.66±1.45	21.85±1.06	34.75±0.85
23	Phenylalanine	2.20±0.13	3.85±0.26	4.66±0.43	3.42±0.16	4.04±0.14	2.48±0.27	2.94±0.21
24	Asparagine	16.18±0.14	11.57±0.16	1.73±0.41	8.01±0.76	11.77±0.60	1.55±0.16	3.36±0.63
26	Citric acid	575.11±5.52	600.89±12.40	501.68±19.10	548.09±36.34	701.75±30.24	438.36±17.31	550.57±5.65
28	Galactose	1.09±0.25	0.40±0.69	1.08±0.10	1.14±0.15	0.57±0.52	ND	1.44±0.01
33	Inositol	9.16±0.44	9.52±0.92	7.31±0.38	14.86±0.59	6.47±0.14	6.57±0.33	8.35±0.11
34	Tryptophan	15.45±3.70	39.36±2.12	18.81±4.61	25.03±1.02	7.85±0.26	15.46±1.39	17.34±5.34
35	Sucrose	312.87±8.91	196.47±6.72	216.15±8.87	225.15±8.31	192.83±3.43	452.87±31.53	273.73±11.65

[a] CI, compound index

[b] ND, not detected

Table S4. Composition and content (ratio/g) of hydrophilic compounds by using non-targeted metabolites profiling, which matched with targeted metabolite profiling, of adzuki bean (*Vigna angularis*) cultivated in China

CI[a]	Compound	C1	C2	C3	C4	C5	C6
1	Lactic acid	12.08±3.99	12.36±2.67	6.64±3.12	6.52±2.26	6.10±1.49	5.02±1.09
6	Urea	5.07±1.19	3.40±0.10	5.99±0.94	10.81±0.90	4.28±0.54	4.60±0.25
7	Serine	3.10±0.61	1.33±1.15	1.82±1.58	17.43±1.06	3.10±0.44	2.62±0.58
8	Ethanolamine	ND[b]	ND	0.58±1.01	2.11±0.11	2.84±0.10	0.90±0.78
10	Isoleucine	8.00±1.80	8.29±0.83	4.83±4.90	12.16±1.41	10.93±0.24	4.92±0.32
11	Proline	5.90±0.22	4.49±0.46	6.76±0.90	14.95±0.62	7.80±0.48	6.42±0.28
12	Glycine	9.76±0.84	9.59±0.37	10.35±0.50	8.83±0.74	9.69±0.16	11.54±0.68
13	Succinic acid	0.67±0.59	1.31±1.14	1.18±1.16	2.59±0.35	2.22±0.11	1.13±0.18
18	Malic acid	36.66±0.91	49.55±1.91	56.08±1.48	29.66±1.72	57.28±4.47	35.64±2.69
19	Aspartic acid	27.89±3.22	23.42±1.90	30.68±3.15	32.66±2.21	27.16±0.94	22.53±0.61
21	Threonic acid	1.76±0.21	1.41±0.05	1.65±0.03	1.47±0.18	1.41±0.17	1.59±0.05
22	Glutamic acid	41.80±2.60	26.73±1.88	35.92±2.11	46.42±2.61	38.97±0.32	34.31±1.76
23	Phenylalanine	2.75±0.20	1.63±1.43	1.73±1.50	2.38±0.28	2.35±0.20	3.57±0.11
24	Asparagine	12.73±1.23	3.44±0.55	6.80±0.54	10.21±0.38	7.75±1.25	13.14±1.23
26	Citric acid	543.33±12.29	489.22±28.12	432.49±12.36	345.73±23.60	391.04±16.51	454.05±37.16
28	Galactose	0.29±0.50	0.61±0.54	1.40±0.11	2.10±0.23	1.80±0.12	0.72±0.63
33	Inositol	6.08±0.51	6.37±0.65	10.44±0.80	24.32±1.01	9.55±0.56	6.54±0.50
34	Tryptophan	37.49±4.60	15.51±1.06	7.29±2.76	5.15±0.14	13.57±1.42	17.65±1.19
35	Sucrose	215.48±13.32	280.74±16.33	351.99±19.97	304.23±12.53	312.81±6.59	163.35±9.19

[a] CI, compound index

[b] ND, not detected

Table S5. OPLS-DA loading and VIP of variables from targeted metabolite profiling with UV-scaling.

Metabolite name	CI[a]	MSI Lv.[b]	OPLS 1	OPLS 2	VIP
Lactic acid	1	Lv. 1	0.106342	-0.04487	0.618997
Alanine	2	Lv. 1	0.03682	0.247723	0.932848
Oxalic acid	3	Lv. 1	0.047531	-0.04855	0.320792
Glycolic acid	4	Lv. 1	-0.02809	0.148835	0.568816
Valine	5	Lv. 1	-0.0149	0.261748	0.964813
Urea	6	Lv. 1	-0.04564	0.25675	0.976994
Serine	7	Lv. 1	-0.09816	0.075711	0.616975
Ethanolamine	8	Lv. 1	0.174015	0.245597	1.32916
Phosphoric acid	9	Lv. 1	0.028024	0.124504	0.483484
Isoleucine	10	Lv. 1	0.003788	0.216767	0.796292
Proline	11	Lv. 1	0.035277	0.171144	0.658902
Glycine	12	Lv. 1	0.139781	0.282461	1.30038
Succinic acid	13	Lv. 1	-0.11368	-0.14653	0.834493
Glyceric acid	14	Lv. 1	-0.16602	0.062191	0.959069
Fumaric acid	15	Lv. 1	0.175265	0.173546	1.17182
Threonine	16	Lv. 1	-0.16112	0.074106	0.944081
β-Alanine	17	Lv. 1	0.126167	0.116685	0.827479
Malic acid	18	Lv. 1	-0.16833	-0.17331	1.13887
Aspartic acid	19	Lv. 1	-0.21853	0.188989	1.40891
Pyroglutamic acid	20	Lv. 1	0.176867	0.059258	1.01595
Threonic acid	21	Lv. 1	-0.13672	0.045836	0.785382
Glutamic acid	22	Lv. 1	-0.15274	0.192444	1.11077
Phenylalanine	23	Lv. 1	-0.30755	-0.07568	1.74784
Asparagine	24	Lv. 1	-0.01533	0.131616	0.490909
Shikimic acid	25	Lv. 1	0.25839	-0.17737	1.58936
Citric acid	26	Lv. 1	-0.32861	-0.04269	1.85041
Fructose	27	Lv. 1	-0.06119	-0.09151	0.480421
Galactose	28	Lv. 1	-0.05832	-0.1739	0.717562
Glucose	29	Lv. 1	-0.10866	-0.14552	0.810715
Mannitol	30	Lv. 1	0.064955	0.067613	0.440988
Lysine	31	Lv. 1	-0.09784	0.201726	0.922015
Tyrosine	32	Lv. 1	-0.10108	0.220439	0.988402
Inositol	33	Lv. 1	0.120004	0.27874	1.22519
Tryptophan	34	Lv. 1	-0.15403	-0.01806	0.866771
Sucrose	35	Lv. 1	0.121299	0.013564	0.682407
Raffinose	36	Lv. 1	0.088481	0.262508	1.0843

[a] CI, compound index

[b] MSI Lv., metabolomics standard initiative level

Table S6. OPLS-DA loading and VIP of variables from non-targeted metabolite profiling with UV-scaling

Metabolite name	MT[a]	CI[b]	MSI Lv.[c]	OPLS 1	OPLS 2	VIP 1
Lactic acid	T[d]	1	Lv. 1	0.051864	-0.01266	0.478799
Urea	T	6	Lv. 1	-0.0632	0.161111	1.15128
Serine	T	7	Lv. 1	-0.03756	0.105895	0.739257
Ethanolamine	T	8	Lv. 1	0.093433	0.178195	1.39292
Isoleucine	T	10	Lv. 1	0.010079	0.092045	0.57698
Proline	T	11	Lv. 1	0.018915	0.162543	1.02056
Glycine	T	12	Lv. 1	0.095095	0.184213	1.4317
Succinic acid	T	13	Lv. 1	-0.06821	-0.04405	0.678382
Malic acid	T	18	Lv. 1	-0.12352	-0.07121	1.2082
Aspartic acid	T	19	Lv. 1	-0.11514	0.046174	1.08691
Threonic acid	T	21	Lv. 1	-0.05284	0.027222	0.509842
Glutamic acid	T	22	Lv. 1	-0.05492	0.036623	0.549175
Phenylalanine	T	23	Lv. 1	-0.16582	-0.06387	1.56108
Asparagine	T	24	Lv. 1	0.034713	0.017721	0.334628
Citric acid	T	26	Lv. 1	-0.19944	-0.08729	1.895
Galactose	T	28	Lv. 1	0.143397	0.143125	1.57802
Inositol	T	33	Lv. 1	0.044777	0.21265	1.37775
Tryptophan	T	34	Lv. 1	-0.0666	-0.10178	0.874443
Sucrose	T	35	Lv. 1	0.045546	0.05207	0.525271
Butanedioic acid	A[e]	37	Lv. 2	-0.02937	-0.12602	0.824444
Ethylene glycol	A	38	Lv. 2	0.104401	-0.01054	0.953066
1,3-Propanediol	A	39	Lv. 2	-0.00221	-0.13361	0.827105
Propane	A	40	Lv. 2	0.107096	-0.08417	1.10576
Hydroxylamine	A	41	Lv. 2	0.076587	-0.06343	0.800393
N,O,O-tri(trimethylsilyl) -1-c(13)-N-carboxy-glycine	A	42	Lv. 2	0.032642	-0.04597	0.4115
1,4-Butanediol	A	43	Lv. 2	0.047661	-0.0104	0.438814
Norvaline	A	44	Lv. 2	0.001568	0.159663	0.988193
Glycerol	A	45	Lv. 2	0.026429	0.188508	1.19118
5-Methoxy-3-chromanol	A	46	Lv. 2	0.13129	0.011624	1.19789
Propanoic acid	A	47	Lv. 2	0.138516	-0.00673	1.26222
Pipecolic acid	A	48	Lv. 2	-0.09873	0.084176	1.03917
L-Aspartic acid	A	49	Lv. 2	-0.0289	-0.01464	0.278336
D-(-)-Erythrose	A	50	Lv. 2	0.195692	-0.03517	1.79551
2-Methoxy-3-methoxy carbonyl-1,3-pentadiene	A	51	Lv. 2	0.117786	0.068522	1.15351
Suberylglycine	A	52	Lv. 2	-0.00195	-0.03158	0.196215
L-(+)-Tartaric acid	A	53	Lv. 2	-0.06516	-0.07279	0.745019
D-Ribose	A	54	Lv. 2	-0.02257	-0.02998	0.276922
D-Galactose	A	55	Lv. 2	-0.10794	-0.07722	1.09303
Allantoin	A	56	Lv. 2	-0.07867	-0.02631	0.734773
L-Histidine	A	57	Lv. 2	-0.12547	-0.11073	1.33246
3-(2-fluorophenyl)spiro [oxirane-2,2'-norbornane]	A	58	Lv. 2	-0.07862	0.060432	0.807857
D-Pinitol	A	59	Lv. 2	-0.01889	0.018008	0.204951

(*Continued next page*)

(*Table S6, continued*)

Metabolite name	MT[a]	CI[b]	MSI Lv.[c]	OPLS 1	OPLS 2	VIP 1
D-(+)-Talopyranose	A	60	Lv. 2	-0.13858	-0.00992	1.26359
Ribonic acid, 2,3,4,5-tetrakis-O-(trimethylsilyl)-, trimethylsilyl ester	A	61	Lv. 2	0.072576	0.202773	1.41832
Galacturonic acid	A	62	Lv. 2	-0.07406	-0.024	0.690639
Pentadecanoic acid	A	63	Lv. 2	0.031247	0.070168	0.519189
Tris(trimethylsilyl)amine	A	64	Lv. 2	-0.02363	-0.08724	0.581214
Ethyl D-glucopyranoside	A	65	Lv. 2	0.142101	-0.00383	1.2944
Cyclohexanecarboxylic acid	A	66	Lv. 2	0.043722	-0.12444	0.866949
Bis(2-ethylhexyl) phthalate	A	67	Lv. 2	-0.07416	-0.06832	0.796828
(2,3-Diphenylcyclopropyl) methyl phenyl sulfoxide1	A	68	Lv. 2	-0.03341	-0.14623	0.954751
(2,3-Diphenylcyclopropyl) methyl phenyl sulfoxide2	A	69	Lv. 2	-0.0536	-0.15586	1.08104
Tetracyclo[4.3.0.0(2,7)] nonane-7,7,8,8-tetracarbonitrile	A	70	Lv. 2	-0.05378	-0.07597	0.67891
Methyl galactoside (1S,2R,3S,4R,5R)-	A	71	Lv. 2	0.101595	-0.01719	0.931373
D-Galactoside	A	72	Lv. 2	0.074345	0.050069	0.744626
D-Glucopyranose	A	73	Lv. 2	0.01459	-0.17575	1.09571
D-Galactose1	A	74	Lv. 2	-0.05193	-0.03828	0.528941
D-Galactose2	A	75	Lv. 2	-0.07834	-0.15395	1.19027
Dimethyl hexopyranosiduronate	A	76	Lv. 2	0.053042	-0.03659	0.533515
Analyte 1	A	A1	Lv. 3	0.070551	-0.06526	0.758919
Analyte 2	A	A2	Lv. 3	0.027076	-0.14568	0.93465
Analyte 3	A	A3	Lv. 3	0.014231	-0.1355	0.848486
Analyte 4	A	A4	Lv. 3	0.169871	0.018552	1.55135
Analyte 5	A	A5	Lv. 3	-0.02621	-0.03166	0.308798
Analyte 6	A	A6	Lv. 3	0.09068	0.127063	1.14035
Analyte 7	A	A7	Lv. 3	0.057617	-0.15033	1.06812
Analyte 8	A	A8	Lv. 3	-0.04199	-0.11182	0.790637
Analyte 9	A	A9	Lv. 3	-0.02519	0.151103	0.962854
Analyte 10	A	A10	Lv. 3	0.075405	-0.06724	0.802995
Analyte 11	A	A11	Lv. 3	0.13748	-0.0253	1.26185
Analyte 12	A	A12	Lv. 3	-0.09436	0.059648	0.935273
Analyte 13	A	A13	Lv. 3	-0.13918	-0.04533	1.29827
Analyte 14	A	A14	Lv. 3	-0.0397	-0.08942	0.661015
Analyte 15	A	A15	Lv. 3	-0.00107	-0.10727	0.663949
Analyte 16	A	A16	Lv. 3	-0.11513	0.060933	1.11432
Analyte 17	A	A17	Lv. 3	-0.10006	-0.04472	0.952433
Analyte 18	A	A18	Lv. 3	0.079012	0.041973	0.765048
Analyte 19	A	A19	Lv. 3	0.045397	-0.13678	0.942076

(Continued next page)

(Table S6, continued)

Metabolite name	MT[a]	CI[b]	MSI Lv.[c]	OPLS 1	OPLS 2	VIP 1
Analyte 20	A	A20	Lv. 3	-0.2268	-0.08097	2.12546
Analyte 21	A	A21	Lv. 3	-0.08346	0.055368	0.833751
Analyte 22	A	A22	Lv. 3	0.100241	-0.10141	1.10786
Analyte 23	A	A23	Lv. 3	0.023844	-0.0808	0.545134
Analyte 24	A	A24	Lv. 3	-0.08741	-0.13484	1.15326
Analyte 25	A	A25	Lv. 3	0.106088	-0.16604	1.41047
Analyte 26	A	A26	Lv. 3	0.075807	0.044955	0.744354
Analyte 27	A	A27	Lv. 3	0.04154	0.016604	0.392035
Analyte 28	A	A28	Lv. 3	0.193454	0.036014	1.77592
Analyte 29	A	A29	Lv. 3	0.021215	-0.10656	0.687177
Analyte 30	A	A30	Lv. 3	-0.09143	-0.05139	0.891395
Analyte 31	A	A31	Lv. 3	0.005233	-0.03018	0.192736
Analyte 32	A	A32	Lv. 3	-0.05484	0.066055	0.645435
Analyte 33	A	A33	Lv. 3	-0.06773	-0.0519	0.695424
Analyte 34	A	A34	Lv. 3	-0.0241	-0.07808	0.530714
Analyte 35	A	A35	Lv. 3	-0.06769	-0.0587	0.715564
Analyte 36	A	A36	Lv. 3	-0.11547	-0.1112	1.25679
Analyte 37	A	A37	Lv. 3	-0.00451	-0.10926	0.677382
Analyte 38	A	A38	Lv. 3	-0.12702	0.052375	1.20138
Analyte 39	A	A39	Lv. 3	-0.0491	-0.118	0.856288
Analyte 40	A	A40	Lv. 3	-0.12389	-0.03641	1.15062
Analyte 41	A	A41	Lv. 3	0.105268	-0.04052	0.990982
Analyte 42	A	A42	Lv. 3	0.152641	-0.04983	1.42398
Analyte 43	A	A43	Lv. 3	0.14626	0.071931	1.40448
Analyte 44	A	A44	Lv. 3	-0.11821	-0.11822	1.30165
Analyte 45	A	A45	Lv. 3	-0.03817	0.003995	0.34851
Analyte 46	A	A46	Lv. 3	-0.02274	-0.1538	0.974086
Analyte 47	A	A47	Lv. 3	0.026273	-0.14998	0.958542
Analyte 48	A	A48	Lv. 3	0.102345	-0.05745	0.997604
Analyte 49	A	A49	Lv. 3	0.141103	-0.04599	1.31624
Analyte 50	A	A50	Lv. 3	-0.01586	-0.02083	0.193614
Analyte 51	A	A51	Lv. 3	0.025712	-0.07669	0.529219
Analyte 52	A	A52	Lv. 3	-0.04446	-0.12557	0.876273

[a] MT, metabolite profiling type
[b] CI, compound index
[c] MSI Lv., metabolomics standard initiative level
[d] T, identified compounds by targeted metabolite profiling
[e] A, automatically identified compounds by non-targeted metabolite profiling

Table S7. OPLS-DA loading and VIP of variables from targeted metabolite profiling with pareto-scaling.

Metabolite name	CI[a]	MSI Lv.[b]	OPLS 1	OPLS 2	VIP 1
Lactic acid	1	Lv. 1	-0.00166	-0.05611	0.196537
Alanine	2	Lv. 1	0.066953	0.004189	0.328412
Oxalic acid	3	Lv. 1	-6.48E-05	0.059424	0.207978
Glycolic acid	4	Lv. 1	0.033658	0.031306	0.198009
Valine	5	Lv. 1	0.024797	-0.00131	0.121595
Urea	6	Lv. 1	0.008847	0.026914	0.103695
Serine	7	Lv. 1	-0.01182	0.029176	0.11739
Ethanolamine	8	Lv. 1	0.134906	0.035028	0.67234
Phosphoric acid	9	Lv. 1	0.084447	0.489669	1.76304
Isoleucine	10	Lv. 1	0.021918	0.005705	0.109244
Proline	11	Lv. 1	0.072573	-0.01804	0.361184
Glycine	12	Lv. 1	0.09799	0.033229	0.494056
Succinic acid	13	Lv. 1	-0.01523	-0.00137	0.074778
Glyceric acid	14	Lv. 1	-0.01579	-0.02374	0.113549
Fumaric acid	15	Lv. 1	0.027061	0.000993	0.13265
Threonine	16	Lv. 1	-0.02	-0.00834	0.102278
β-Alanine	17	Lv. 1	0.016614	-0.01719	0.101223
Malic acid	18	Lv. 1	-0.24132	0.165889	1.31735
Aspartic acid	19	Lv. 1	-0.08129	-0.04363	0.42663
Pyroglutamic acid	20	Lv. 1	0.05981	-0.07401	0.39113
Threonic acid	21	Lv. 1	-0.00404	-0.02915	0.103934
Glutamic acid	22	Lv. 1	-0.04621	-0.05543	0.298156
Phenylalanine	23	Lv. 1	-0.03545	-0.03386	0.210294
Asparagine	24	Lv. 1	0.020858	-0.07794	0.291289
Shikimic acid	25	Lv. 1	0.01603	0.000765	0.078596
Citric acid	26	Lv. 1	-0.89757	-0.50135	4.7354
Fructose	27	Lv. 1	0.017249	0.034306	0.146834
Galactose	28	Lv. 1	-0.02441	-0.00611	0.121496
Glucose	29	Lv. 1	-0.01485	0.003973	0.074068
Mannitol	30	Lv. 1	0.003067	0.000966	0.015403
Lysine	31	Lv. 1	-0.01337	-0.00553	0.068304
Tyrosine	32	Lv. 1	-0.01476	0.01837	0.096763
Inositol	33	Lv. 1	0.102869	0.037753	0.521113
Tryptophan	34	Lv. 1	-0.10894	-0.07205	0.590378
Sucrose	35	Lv. 1	-0.08918	0.664626	2.36681
Raffinose	36	Lv. 1	0.173234	0.003957	0.848999

[a] CI, compound index

[b] MSI Lv., metabolomics standard initiative level

Table S8. OPLS-DA loading and VIP of variables from non-targeted metabolite profiling with pareto-scaling

Metabolite name	MT[a]	CI[b]	MSI Lv.[c]	OPLS 1	OPLS 2	VIP 1
Lactic acid	T[d]	1	Lv. 1	-0.0216	-0.03488	0.283015
Urea	T	6	Lv. 1	-0.01072	0.036714	0.242736
Serine	T	7	Lv. 1	0.004458	-0.01047	0.074674
Ethanolamine	T	8	Lv. 1	0.109225	0.065939	1.02375
Isoleucine	T	10	Lv. 1	0.008351	0.020336	0.143657
Proline	T	11	Lv. 1	0.042337	0.023998	0.393151
Glycine	T	12	Lv. 1	0.085531	0.034745	0.766887
Succinic acid	T	13	Lv. 1	-0.03428	0.020736	0.321381
Malic acid	T	18	Lv. 1	-0.25134	0.143312	2.33595
Aspartic acid	T	19	Lv. 1	-0.08014	-0.08763	0.873986
Threonic acid	T	21	Lv. 1	-0.00414	-0.00723	0.056812
Glutamic acid	T	22	Lv. 1	-0.00372	-0.11836	0.724427
Phenylalanine	T	23	Lv. 1	-0.0323	-0.03482	0.350402
Asparagine	T	24	Lv. 1	0.047543	-0.09193	0.695516
Citric acid	T	26	Lv. 1	-0.77563	-0.57343	7.54625
Galactose	T	28	Lv. 1	0.024298	0.036061	0.304036
Inositol	T	33	Lv. 1	0.087718	0.070135	0.868911
Tryptophan	T	34	Lv. 1	-0.0641	-0.11676	0.902569
Sucrose	T	35	Lv. 1	-0.11978	0.612162	3.8827
Butanedioic acid	A	37	Lv. 2	-0.0314	-0.01022	0.277611
Ethylene glycol	A	38	Lv. 2	0.029306	-0.00972	0.259382
1,3-Propanediol	A	39	Lv. 2	-0.07024	-0.0646	0.722677
Propane	A	40	Lv. 2	0.026729	0.065833	0.46375
Hydroxylamine	A	41	Lv. 2	0.008733	-0.02792	0.186544
N,O,O-tri(trimethylsilyl) -1-c(13)-N-carboxy-glycine	A	42	Lv. 2	0.049055	0.023371	0.44613
1,4-Butanediol	A	43	Lv. 2	-0.0116	-0.01275	0.126755
Norvaline	A	44	Lv. 2	0.017415	-0.00013	0.150036
Glycerol	A	45	Lv. 2	0.021154	0.046498	0.337704
5-Methoxy-3-chromanol	A	46	Lv. 2	0.005207	0.033749	0.211178
Propanoic acid	A	47	Lv. 2	0.009415	0.030487	0.203295
Pipecolic acid	A	48	Lv. 2	-0.17265	-0.13566	1.70312
L-Aspartic acid	A	49	Lv. 2	-0.00364	-0.00117	0.032142
D-(-)-Erythrose	A	50	Lv. 2	0.01901	0.005782	0.167548
2-Methoxy-3-methoxy carbonyl-1,3-pentadiene	A	51	Lv. 2	0.065919	-0.04513	0.631387
Suberylglycine	A	52	Lv. 2	-0.00281	-0.00623	0.045172
L-(+)-Tartaric acid	A	53	Lv. 2	-0.03109	-0.14922	0.950894
D-Ribose	A	54	Lv. 2	0.014142	0.022936	0.185773
D-Galactose	A	55	Lv. 2	-0.02736	0.006438	0.238947
Allantoin	A	56	Lv. 2	-0.02163	-0.05943	0.408371
L-Histidine	A	57	Lv. 2	-0.05534	-0.1211	0.880679
3-(2-fluorophenyl)spiro [oxirane-2,2'-norbornane]	A	58	Lv. 2	-0.01409	-0.00019	0.121414
D-Pinitol	A	59	Lv. 2	0.06177	-0.07727	0.711653

(*Continued next page*)

(*Table S8, continued*)

Metabolite name	MT[a]	CI[b]	MSI Lv.	OPLS 1	OPLS 2	VIP 1
D-(+)-Talopyranose	A	60	Lv. 2	-0.02341	-0.0012	0.201818
Ribonic acid, 2,3,4,5-tetrakis-O-(trimethylsilyl)-, trimethylsilyl ester	A	61	Lv. 2	0.059434	0.035899	0.557105
Galacturonic acid	A	62	Lv. 2	-0.14743	0.12798	1.49183
Pentadecanoic acid	A	63	Lv. 2	-0.00747	0.034847	0.222587
Tris(trimethylsilyl)amine	A	64	Lv. 2	-0.0785	-0.08667	0.85923
Ethyl D-glucopyranoside	A	65	Lv. 2	0.061589	-0.03732	0.577602
Cyclohexanecarboxylic acid	A	66	Lv. 2	-0.01628	-0.04479	0.307694
Bis(2-ethylhexyl) phthalate	A	67	Lv. 2	-0.16374	0.020172	1.41606
(2,3-Diphenylcyclopropyl) methyl phenyl sulfoxide1	A	68	Lv. 2	-0.03493	-0.02873	0.348434
(2,3-Diphenylcyclopropyl) methyl phenyl sulfoxide2	A	69	Lv. 2	-0.04273	-0.04749	0.468876
Tetracyclo[4.3.0.0(2,7)] nonane-7,7,8,8-tetracarbonitrile	A	70	Lv. 2	-0.02062	-0.00527	0.180534
Methyl galactoside (1S,2R,3S,4R,5R)-	A	71	Lv. 2	0.02624	0.001733	0.226316
D-Galactoside	A	72	Lv. 2	0.047089	0.008429	0.408949
D-Glucopyranose	A	73	Lv. 2	-0.12125	-0.18603	1.54439
D-Galactose1	A	74	Lv. 2	-0.20828	0.04223	1.81291
D-Galactose2	A	75	Lv. 2	-0.10397	-0.00176	0.895806
Dimethyl hexopyranosiduronate	A	76	Lv. 2	0.023005	-0.11856	0.751504
Analyte 1	A	A1	Lv. 3	0.000837	-0.03898	0.238431
Analyte 2	A	A2	Lv. 3	-0.01697	-0.01198	0.163544
Analyte 3	A	A3	Lv. 3	-0.03227	-0.03816	0.362906
Analyte 4	A	A4	Lv. 3	0.037834	-0.0026	0.326342
Analyte 5	A	A5	Lv. 3	-0.00559	0.001226	0.048736
Analyte 6	A	A6	Lv. 3	0.03851	-0.00557	0.333522
Analyte 7	A	A7	Lv. 3	-0.00316	-0.02032	0.127176
Analyte 8	A	A8	Lv. 3	-0.02367	-0.01136	0.215408
Analyte 9	A	A9	Lv. 3	0.028696	0.043912	0.364979
Analyte 10	A	A10	Lv. 3	0.011865	-0.0099	0.118788
Analyte 11	A	A11	Lv. 3	0.017469	0.014907	0.175951
Analyte 12	A	A12	Lv. 3	-0.00856	0.02413	0.164939
Analyte 13	A	A13	Lv. 3	-0.09701	-0.03666	0.86528
Analyte 14	A	A14	Lv. 3	-0.01533	0.00534	0.136065
Analyte 15	A	A15	Lv. 3	-0.00996	-0.0137	0.119884
Analyte 16	A	A16	Lv. 3	-0.01538	-0.02064	0.182968
Analyte 17	A	A17	Lv. 3	-0.01922	-0.02118	0.210256
Analyte 18	A	A18	Lv. 3	0.014929	-0.01372	0.153568
Analyte 19	A	A19	Lv. 3	-0.00234	-0.01556	0.09728
Analyte 20	A	A20	Lv. 3	-0.06368	-0.05519	0.644079
Analyte 21	A	A21	Lv. 3	-0.01147	0.016338	0.140487
Analyte 22	A	A22	Lv. 3	0.011625	-0.02658	0.1909

(Continued next page)

(Table S8, continued)

Metabolite name	MT[a]	CI[b]	MSI Lv.	OPLS 1	OPLS 2	VIP 1
Analyte 23	A	A23	Lv. 3	-0.0078	-0.05668	0.353012
Analyte 24	A	A24	Lv. 3	-0.0199	-0.03386	0.268799
Analyte 25	A	A25	Lv. 3	-0.00121	-0.01555	0.095665
Analyte 26	A	A26	Lv. 3	0.01756	-0.00316	0.152514
Analyte 27	A	A27	Lv. 3	0.002775	-0.02677	0.165412
Analyte 28	A	A28	Lv. 3	0.104467	-0.02121	0.909304
Analyte 29	A	A29	Lv. 3	0.005616	-0.00504	0.05736
Analyte 30	A	A30	Lv. 3	-0.06056	-0.07369	0.689399
Analyte 31	A	A31	Lv. 3	0.00123	-0.00909	0.056555
Analyte 32	A	A32	Lv. 3	-0.00818	-0.01565	0.118851
Analyte 33	A	A33	Lv. 3	-0.04041	0.025715	0.381996
Analyte 34	A	A34	Lv. 3	-0.00255	0.013494	0.085375
Analyte 35	A	A35	Lv. 3	-0.04127	0.029346	0.398256
Analyte 36	A	A36	Lv. 3	-0.0405	-0.02406	0.37866
Analyte 37	A	A37	Lv. 3	-0.00512	-0.00957	0.073258
Analyte 38	A	A38	Lv. 3	-0.04844	-0.01184	0.423528
Analyte 39	A	A39	Lv. 3	-0.07016	0.049926	0.677149
Analyte 40	A	A40	Lv. 3	-0.04136	-0.02417	0.385781
Analyte 41	A	A41	Lv. 3	0.011704	0.007672	0.111212
Analyte 42	A	A42	Lv. 3	0.041715	-0.03414	0.415621
Analyte 43	A	A43	Lv. 3	0.016378	0.008864	0.151155
Analyte 44	A	A44	Lv. 3	-0.0187	-0.01374	0.181693
Analyte 45	A	A45	Lv. 3	-0.0073	0.001802	0.06381
Analyte 46	A	A46	Lv. 3	0.018513	0.014666	0.182972
Analyte 47	A	A47	Lv. 3	-0.01307	-0.02838	0.20685
Analyte 48	A	A48	Lv. 3	-0.03326	0.0906	0.623695
Analyte 49	A	A49	Lv. 3	0.011804	0.012121	0.125834
Analyte 50	A	A50	Lv. 3	0.01118	-0.01593	0.136965
Analyte 51	A	A51	Lv. 3	-0.00464	0.019497	0.125746
Analyte 52	A	A52	Lv. 3	-0.13772	0.084567	1.29424

[a] MT, metabolite profiling type
[b] CI, compound index
[c] MSI Lv., metabolomics standard initiative level
[d] T, identified compounds by targeted metabolite profiling
[e] A, automatically identified compounds by non-targeted metabolite profiling

Table S9. Relative retention times (RRT) and mass spectral data of hydrophilic compounds as rimethylsilyl derivative.

Compound	RT[a]	RRT[b]	Quantification ion[c]	Mass fragment ion[d]
Lactic acid	04:12.9	0.409	147	117, 147, 191
Alanine	04:42.9	0.458	116	116, 147, 190
Oxalic acid	05:08.9	0.500	147	147
Glycolic acid	05:49.8	0.566	147	147, 177, 205
Valine	05:56.5	0.577	144	144, 156, 218
Urea	06:13.7	0.605	189	147, 171, 189
Serine (1)	06:22.5	0.619	116	116, 132, 147
Ethanolamine	06:27.8	0.628	174	100, 147, 174
Phosphoric acid	06:29.4	0.630	299	299
Isoleucine	06:43.4	0.653	158	147, 158, 218
Proline	06:47.6	0.660	142	142, 158, 216
Glycine	06:51.5	0.666	174	147, 174, 248
Succinic acid	06:56.3	0.674	147	129, 147, 247
Glyceric acid	07:02.8	0.684	147	133, 147, 189
Fumaric acid	07:16.9	0.707	245	143, 147, 245
Serine (2)	07:20.4	0.713	204	204, 278, 306
Threonine	07:34.2	0.735	219	117, 218, 291
β-Alanine	07:59.0	0.775	174	147, 174, 248
Malic acid	08:28.1	0.823	147	147, 233, 245
Aspartic acid	08:44.7	0.849	100	100, 147, 232
Pyroglutamic acid	08:49.0	0.856	156	147, 156, 230
Threonic acid	09:01.7	0.877	147	147, 205, 220
Glutamic acid	09:32.9	0.927	246	128, 156, 246
Phenylalanine	09:39.4	0.938	218	100, 192, 218
Asparagine	09:56.6	0.966	116	116, 132, 231
Ribitol (IS)	10:17.7	1.000	217	103, 147, 217
Shikimic acid	10:55.2	1.061	204	147, 204, 255
Citric acid	11:00.1	1.069	273	147, 273, 347
Fructose (1)	11:19.8	1.101	103	103, 147, 217
Fructose (2)	11:23.8	1.107	103	103, 147, 217
Galactose	11:30.1	1.117	147	147, 205, 319
Glucose	11:30.4	1.118	147	147, 160, 205
Mannitol	11:42.9	1.138	319	147, 217, 319
Lysine	11:43.4	1.139	174	174, 317
Tyrosine	11:51.7	1.152	218	218, 280
Inositol	12:47.3	1.242	305	147, 217, 305
Tryptophan	13:37.1	1.323	202	202, 219, 348
Sucrose	15:44.6	1.529	217	147, 217, 361
Raffinose	19:10.1	1.862	217	204, 217, 361

[a] RT, retention time (min:sec)

[b] RRT, relative retention time (retention time of the analyte/retention time of the IS)

[c] Quantification ion, Specific mass ion for quantification

[d] Mass fragment ion, Ions in boldface indicate the specific mass ion used for quantification
